# Simultaneous EEG-fMRI in Patients with Unverricht-Lundborg Disease: Event-Related Desynchronization/Synchronization and Hemodynamic Response Analysis

**DOI:** 10.1155/2010/164278

**Published:** 2010-01-06

**Authors:** Elisa Visani, Ludovico Minati, Laura Canafoglia, Isabella Gilioli, Lucia Salvatoni, Giulia Varotto, Patrik Fazio, Domenico Aquino, Maria Grazia Bruzzone, Silvana Franceschetti, Ferruccio Panzica

**Affiliations:** ^1^Department of Neurophysiopathology, “Carlo Besta” IRCCS Neurological Institute, 20133 Milan, Italy; ^2^Department of Neuroradiology, “Carlo Besta” IRCCS Neurological Institute, 20133 Milan, Italy; ^3^Scientific Department, “Carlo Besta” IRCCS Neurological Institute, 20133 Milan, Italy

## Abstract

We performed simultaneous acquisition of EEG-fMRI in seven patients with Unverricht-Lundborg disease (ULD) and in six healthy controls using self-paced finger extension as a motor task. The event-related desynchronization/synchronization (ERD/ERS) analysis showed a greater and more diffuse alpha desynchronization in central regions and a strongly reduced post-movement beta-ERS in patients compared with controls, suggesting a significant dysfunction of the mechanisms regulating active movement and movement end. The event-related hemodynamic response obtained from fMRI showed delayed BOLD peak latency in the contralateral primary motor area suggesting a less efficient activity of the neuronal populations driving fine movements, which are specifically impaired in ULD.

## 1. Introduction

The analysis of the EEG recorded during motor performance (self-paced movement) provides information about the movement-related changes in oscillatory cortical activity. In normal subjects, an amplitude attenuation of specific frequency components (event-related desynchronization, ERD) in the *α*- and *β*-bands precedes a voluntary movement and reflects cortical activation concurring with movement planning. At the end of the movement, event-related synchronization (ERS) in the *β*-band replaces ERD [1]. Simultaneous EEG-fMRI acquisition during performance of a motor task enables the identification of changes of brain activity in motor areas and provides information on the source of the event generator. 

In Unverricht-Lundborg disease (ULD) patients, voluntary movements are selectively impaired by the presence of action myoclonus [2]. In these patients, ERD/ERS changes highlight increased and diffuse activation of the motor cortex during movement planning and severely reduced postexcitatory inhibition of the motor cortex [3].

We simultaneously acquired EEG and fMRI in order to study the spatiotemporal pattern of ERD/ERS resulting from self-paced extension of the index finger in ULD patients and to explore the correlation with hemodynamic changes.

## 2. Material and Methods

We enrolled 7 right-handed patients (mean age: 29.1 ± 10 years; four women) with ULD, whose main clinical features are reported in Table 1 and 6 right-handed healthy controls (mean age: 29.1 ± 6.7 years; five women). In all patients, the diagnosis of ULD was established on the basis of the typical electroclinical presentation and of the genetic finding of dodecamer expansion at *cstb* gene [4].

### 2.1. Motor Task

Inside the bore of the scanner, subjects laid supine with their arms relaxed; their head was stabilized with adjustable padded restraints on both sides. They were instructed to remain as still as possible throughout the experiment, to keep their eyes open and avoid blinking during the task. Subjects were asked to perform brisk (i.e., lasting less than one second) self-paced extensions of the right index, with a time interval between the end of a movement and the onset of the following one of about 10 seconds. Each subject was trained for several minutes before the experiment. The movement was monitored by electromyography (EMG) and visual observation.

### 2.2. EEG-fMRI Acquisition

EEG was acquired using an MR compatible EEG amplifier (SD MRI 32, Micromed, Treviso, Italy) and a cap providing 30 Ag/AgCl electrodes positioned according to the 10/20 system. Impedance was kept below 5 kΩ. Electrocardiogram (ECG) and EMG were simultaneously recorded. The EMG activity was recorded from pairs of Ag/AgCl surface electrodes placed bilaterally 2–3 cm apart over the right index flexor muscles. EEG data were acquired at the rate of 1024 Hz using the software package provided by the manufacturer.

Imaging was performed on a 1.5 T MR scanner (Magnetom Avanto, Siemens AG, Erlangen, Germany). Functional images were acquired with an axial gradient-echo echo-planar sequence (21 slices, TR = 2000 ms, TE = 50 ms, 2 × 2 mm^2^ in-plane voxel size, 5 mm slice thickness, no gap). A *T*_1_-weighted anatomical scan (160 slices, TR = 1640 ms, TE = 2 ms; 1 mm^3^ isotropic voxels) was also acquired.

The scanner provided a trigger signal corresponding to the excitation of the first slice of each volume, which was recorded by the EEG system enabling real-time artefact removal, making possible to monitor the EEG signal as well as task performance through EMG.

### 2.3. Data Analysis

The imaging gradient artefact and the ballistocardiogram were digitally removed from the EEG using an adaptive filter [6], implemented on software provided by the manufacturer.

Movement onset was determined by the beginning of the burst of EMG activity. EEG data were epoched four seconds before and three seconds after movement onset. Epochs with artefacts, incomplete muscle relaxation between movements, and intertrial interval shorter than 8 seconds were excluded from the analysis. A reference period at rest, from 3500 to 2500 ms before movement onset, was considered. Each trial was digitally band-pass filtered from 1 Hz below to 1 Hz above the individual frequencies of the most movement-sensitive power peaks in *α*- and *β*-bands. The filtered EEG data were then squared and averaged over all trials and over time (one value every 125 ms). The ERD or ERS values were calculated according to the following formula:

(1) $$\text{ERD}/S\left(k\right)=\frac{A\left(k\right)-R}{R}\times 100,$$

where *A*(*k*) is the power at sample *k* and *R* represents the mean power of the reference period (negative values correspond to ERD, positive values to ERS).

The statistical significance of the differences between the mean power observed during the reference period and that measured during the subsequent 125-milisecond intervals was expressed as a probability value using Wilcoxon's signed rank test. The power changes were considered significant when the *P* value was less than .05. ERD/ERS data analysis was performed using software developed in Matlab (Mathworks Inc., Natick, MA, USA). For statistical analysis, we divided the time course of ERD/ERS in five epochs of 1 second each (*t*1: −2.5 to −1.5 s, *t*2: −1.5 to −0.5 s, *t*3: − 0.5 to 0.5 s, *t*4:  0.5 to 1.5 s, *t*5:  1.5 to 2.5 s) and we compared the values measured on F4, C4, P4, F3, C3, P3, Fz, Cz, and Pz electrodes.

The fMRI data were analyzed by means of the SPM5 software (Wellcome Neuroimaging Dept., Institute of Neurology, London, UK). Preprocessing included three-dimensional motion correction, slice-timing correction, Gaussian smoothing, and normalization into MNI (Montreal Neurological Institute) space. First-level analysis was performed by general linear model (GLM), using the event function from EMG, convolved with the canonical hemodynamic response function, as regressor. Three-dimensional regions of interest (ROIs) were manually drawn for each subject by an experienced operator on the contralateral and ipsilateral primary motor areas as well as on the contralateral supplementary motor area. The average signal time-course was obtained, and the amplitude and latency of the peak of the fitted hemodynamic response were measured.

For statistical analysis, the Mann-Whitney *U* test was applied.

## 3. Results

All subjects performed the motor task well: the mean movement duration was on average longer in the patients group (535.8 ± 110.3 versus 728.6 ± 195.5 ms; *P* = .062). The *α*- and *β*-band peak frequencies, selected as movement reactive EEG frequency, were lower in the patient group with respect to controls and in *α*-band this difference reached statistical significance (*α*: 11.3 ± 0.8 versus 9.1 ± 1.6 Hz, *P* = .02;  *β*: 22 ± 5.6 versus 18.6 ± 1.5 Hz).

### 3.1. ERD/ERS Analysis

**Figure 1:**
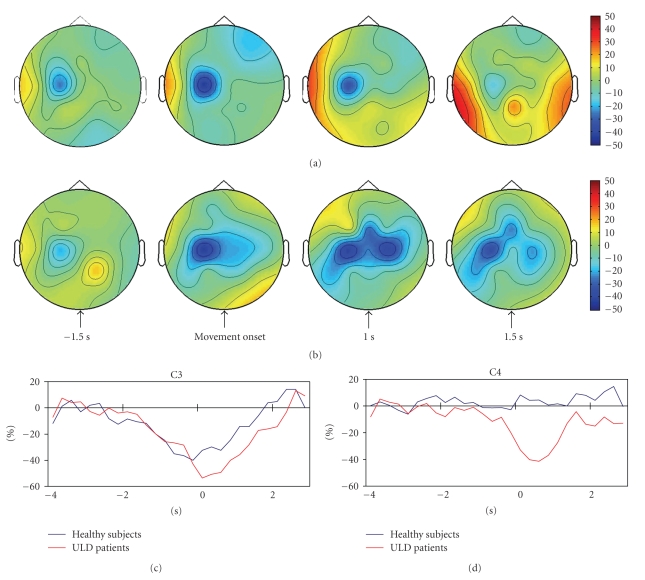
Color maps showing the grand average of *α*-ERD in control subjects (a) and in patients (b). Color scale: maximum ERD and ERS are coded in blue and red. The lower panels show the grand average of *α*-ERD time series recorded from contralateral (c) and ipsilateral (d) central derivations in patients and controls.

In all subjects *α*- and *β*-ERD were observed (Figure 1). The time course of the *α*- and *β*-desynchronization was similar for the two groups, but in patients the desynchronization in the *α*-band was significantly greater in the contralateral central derivation (−48.5 ± 13 versus −58.4 ± 9.6; *P* = .032, for controls and patients, Figure 1(c)) and also involved the midline and the ipsilateral central derivations (Table 2 and Figures 1(b) and 1(d)).

**Figure 2:**
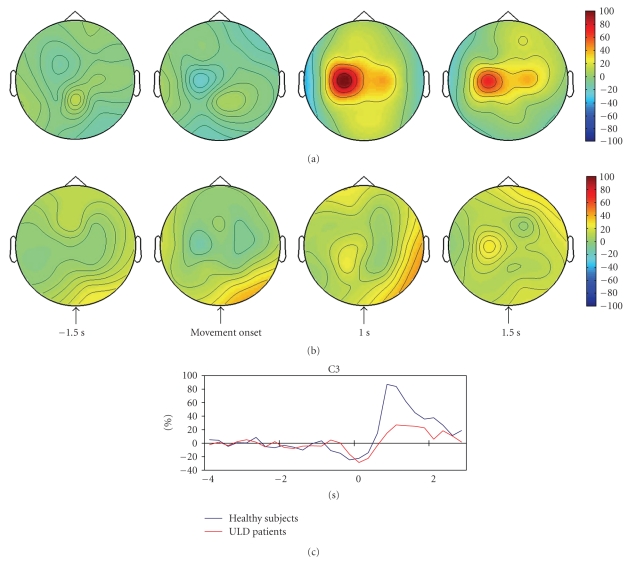
Color maps showing the grand average of *β*-ERD/ERS in control subjects (a) and patients (b). Color scale: maximum ERD and ERS are coded in blue and red. The lower panel (c) shows the grand average of *β*-ERD/ERS time series recorded from the contralateral central derivation in patients and controls.

The expected postmovement *β*-ERS was observed in all controls; it was undetectable in two patients, whereas in the remaining patients it was significantly smaller with respect to that measured in controls (107.5 ± 86.9 versus 31.3 ± 8.8; *P* = .025, for controls and patients, Table 2 and Figure 2).

### 3.2. fMRI Analysis

**Figure 3:**
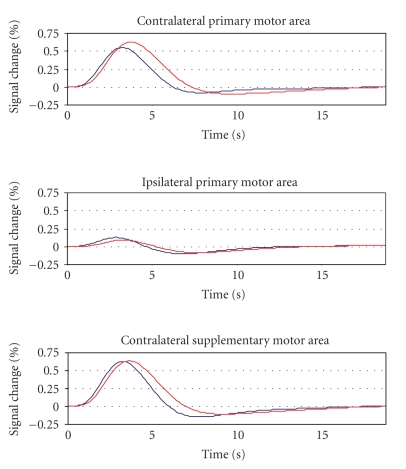
Time-courses of the hemodynamic response for controls (blue) and patients (red).

The peak amplitude of the hemodynamic response was comparable in controls and patients in the contralateral (0.56 ± 0.18% versus 0.63 ± 0.30%, *P* = .6) and ipsilateral (0.17 ± 0.15% versus 0.15 ± 0.14%, *P* = .8) motor areas as well as in the contralateral supplementary motor area (0.58 ± 0.15% versus 0.60 ± 0.17%, *P* = .8). There was, however, a trend towards longer response latency in patients, which reached statistical significance in the contralateral motor area (3.1 ± 0.4 s versus 3.6 ± 0.5 s, *P* = .011) and approached statistical significance in the contralateral supplementary motor area (3.1 ± 0.4 s versus 3.4 ± 0.2 s, *P* = .08); the effect was not found in the ipsilateral motor area (2.7 ± 0.2 s versus 3.3 ± 0.8 s, *P* = .1) (Figure 3).

## 4. Discussion and Conclusions

The changes found in ERD/ERS pattern of ULD subjects suggest an increased activation of motor cortex during movement planning and a significant reduction of post-excitatory inhibition. These data overlap those obtained in our previous study [3] on EEG signal recorded in standard laboratory and demonstrate the applicability of the eventrelated protocol during simultaneous EEG/fMRI.

Differently from ERD/ERS, fMRI did not highlight any clear difference in the amplitude of cortical activation in ULD patient with respect to controls. The hemodynamic response analysis allowed detecting subtle but significant effects on the time course of activation that showed a delayed peak in ULD patients. This finding, together with the slightly longer duration of individual movements in patients with respect to controls, may agree with a less efficient performance of the motor cortex in this disorder, characterized by a prominent motor dysfunction resulting in action activated myoclonic jerks. Based on the present data, the ERD/ERS changes detectable on EEG appear to be more reliable with respect to fMRI in detecting the cortical dysfunction characterizing ULD patients, being able to detect and quantify the functional changes of the neuronal pools impaired by the disease. A final conclusion cannot however be reached because of the small number of observation that limited the statistical power; moreover, further analyses exploring the functional connectivity during motor performance may allow to better detect subtle changes in the BOLD signals [7].

## Figures and Tables

**Table 1:** Patient data.

Subject, Age [yrs], Sex	Disease duration [yrs]	AED	Simplified myoclonus rating
1, 22, f	12	VPA, TPM, CLZ	2
2, 26, f	16	VPA, CZP	2
3, 36, m	22	VPA, LVT, PB	2
4, 25, m	14	VPA, CZP, piracetam	3
5, 49, m	34	VPA, TPM	2
6, 22, f	11	VPA, LVT, TPM	2
7, 24, f	12	VPA	3

AED: antiepileptic drugs; VPA: valproate; TPM: topiramate; CLZ: clobazam; CZP: clonazepam LVT: levetiracetam; PB: phenobarbital. Simplified myoclonous rating [5]: 2: mild myoclonous, interference with fine movements and/or speech, no interference with walking; 3: moderate myoclonous, patient still able to walk without support.

**Table 2:** Statistical analysis of ERD/ERS values assessed in subsequent epochs.

Alpha ERD	F4	C4	P4	F3	C3	P3	Fz	Cz	Pz
*t*1	—	0.015	—	—	—	—	—	0.032	—
*t*2	—	0.010	—	—	—	0.042	—	—	—
*t*3	—	0.003	—	—	—	—	—	0.003	—
*t*4	0.045	0.010	—	—	0.015	0.004	0.007	0.003	0.032
*t*5	—	—	—	—	—	—	—	0.007	—

Beta ERD/ERS*	F4	C4	P4	F3	C3	P3	Fz	Cz	Pz

*t*1	—	—	—	—	—	—	—	—	—
*t*2	—	—	—	—	—	—	—	—	—
*t*3	—	—	—	—	—	—	—	—	—
*t*4	0.032	—	0.007	—	0.032	—	0.015	—	—
*t*5	—	0.032	0.022	—	0.012	—	—	0.010	—

Results of *U*-Mann Whitney test between patients and controls group. —= not significant. *t1-* t*3 correspond to beta ERD and *t*4-*t*5 to beta ERS.
